# Process evaluation of an up-scaled community based child obesity treatment program: NSW Go4Fun®

**DOI:** 10.1186/1471-2458-14-140

**Published:** 2014-02-10

**Authors:** Debra Welsby, Binh Nguyen, Blythe J O’Hara, Christine Innes-Hughes, Adrian Bauman, Louise L Hardy

**Affiliations:** 1 NSW Office of Preventive Health, Liverpool Hospital, Locked Bag 7103, Liverpool BC, Sydney, NSW, Australia; 2 Physical Activity Nutrition Obesity Research Group, Level 2, Medical Foundation Building K25, University of Sydney, Sydney, NSW 2006, Australia

**Keywords:** Translational research, Child obesity, Up-scaling, Process evaluation

## Abstract

**Background:**

Community-based obesity treatment programs for children that have a large program reach are a priority. To date, most programs have been small efficacy trials whose findings have yet to be up-scaled and translated into real-world settings. This paper reports on the process evaluation of a government-funded, translated obesity treatment program for children in Australia. It describes the characteristics and reach of children participating in the New South Wales (NSW) Ministry of Health Go4Fun® program.

**Methods:**

Delivered across the state of NSW (Australia) by Local Health Districts (LHDs), Go4Fun® is a community-based, multidisciplinary family obesity treatment program adapted from the United Kingdom Mind Exercise Nutrition Do it (MEND) program that targets weight-related behaviours. Children aged 7-13 years with a BMI ≥85^th^ percentile and no co-morbidities were eligible at no cost. Parents/carers self-refer via a toll-free phone number, text messages, online registration or via secondary referrals. LHDs deliver a 16 to 20-session program based on length of school term, holidays and recruitment challenges. Both parent/carer and child attend bi-weekly after school sessions. Parent-reported socio-demographic and measured child weight characteristics are presented using descriptive statistics. Differences between completers (attended at least 75% of sessions) and non-completers were assessed using chi-square tests, independent sample t-tests and adjusted odds ratios. Analyses were adjusted for clustering of programs.

**Results:**

Between 2009 and 2012, a total of 2,499 children (54.8% girls; mean age [SD]: 10.2 [1.7 years]) participated in the Go4Fun® program. Children were mainly from low-middle socioeconomic status (76.5%), resided in major cities (63.3%), and 5.7% were Aboriginal. At baseline, 96.5% of children were overweight or obese. Mean BMI-z-score was 2.07 (0.41) and 94.5% had a waist-to-height ratio ≥0.5. More than half (57.9%) completed at least 75% of sessions. Amongst completers (N = 1,446), girls (56.8%; p = 0.02), non-Aboriginal children (95.9%; p < 0.01) and children residing in less socially disadvantaged areas (25.9%; p = 0.02) were significantly more likely to complete the program.

**Conclusions:**

The Go4Fun® program successfully reached the targeted population of overweight/obese children at socioeconomic disadvantage and is a rare example of an up-scaled translational program.

## Background

Childhood obesity is one of Australia’s major public health problems, with implications for current and future health services. In Australia’s most populous state, New South Wales (NSW, population = 7.2 million; 802,000 km^2^), the prevalence of obesity among children aged 7-13 years in 2010 was 6.8% [1], or approximately 42,500 children [2]. Further there are significant socioeconomic disparities in the prevalence of obesity, with higher prevalence occurring in children from low, compared with high socioeconomic backgrounds [3]. Despite the high prevalence and multiple national action plans, tertiary paediatric obesity services in Australia are inadequate to meet treatment requirements of these children [4] and funding for child obesity interventions is limited [5]. To this end, alternative or adjunctive treatment and secondary prevention modalities for child obesity, such as community-based programs that have large population reach, are a priority.

Community-based obesity treatment programs have become an important response to address child obesity, however the majority of these programs are small, feasibility trials and these findings are yet to be up-scaled and translated into real-world context [6]. The purpose of up-scaling public health programs is to improve reach (population and geographical access) and equitable access to a program and its intended benefits [7]. While it is important to translate research findings beyond controlled, small settings into real-world context, it is of potentially greater importance to report on the effectiveness of such up-scaled interventions both in terms of process (implementation) and outcomes (effectiveness) [6].

The United Kingdom Mind Exercise Nutrition Do it (UK MEND) program is an example of a community based child obesity program which has demonstrated efficacy in weight outcomes [8,9] The utility of community based programs is to target groups of individuals, rather than individuals within a particular geographical area to have greater population reach. Furthermore, community-based programs build community capacity by employing local professionals who have greater awareness of their community’s characteristics and vested interest in success. Once programs such as MEND have been evaluated through randomised control trials [9] (i.e. innovation testing) and replicated [10], the next step is testing whether the program can be disseminated (i.e. up-scaling) across a population in a variety of community settings [11]. Accordingly, MEND was designed to be scalable and delivered by a range of health and social care professionals and has now been translated into the NSW context (Go4Fun®) and disseminated across 296 NSW communities and has a particular emphasis to ensure it reaches disadvantaged communities and accordingly, low socioeconomic and regional areas.

Go4Fun has been widely disseminated and therefore process evaluation is now prudent. Information generated from the process evaluation is especially important for building evidence for real-world, up-scaled programs which have different characteristics to small, controlled efficacy programs. This type of information informs policy makers and program deliverers on program strengths, weaknesses, and areas that need improvement. This paper describes the up-scaling of Go4Fun in NSW and the characteristics of the population it has reached and retained since inception in 2009, including the characteristics of children who completed and did not complete the program.

## Methods

### Context

In 2008 the National Partnership Agreement on Preventive Health (NPAPH) was announced by the Australian government providing $AUD932.5 million dollars in funding to strengthen Australia’s investment and infrastructure in preventative health. One of the initiatives under the Agreement is the Healthy Children Initiative (HCI) which delivers programmatic activities to 0-18 year olds promoting healthy weight, healthy eating and physical activity. In NSW, one of the HCI programs is Go4Fun, which is supported and funded by the state-wide Office of Preventive Health and locally implemented by Health Promotion Services of Local Health Districts (LHD). Briefly, the NSW health service is divided geographically into 15 LHD (eight cover the Sydney metropolitan region, and seven cover rural and regional NSW). Program funding is allocated according to the prevalence of child overweight/obesity and geographical spread (km^2^) of the LHD.

Engaging LHD’s for program dissemination utilises the expertise and local knowledge of these services and the extensive existing local relationships with other government departments, non government organisations, clinical services and the community. For example engaging local schools has been a very successful approach, resulting in the greatest proportion of referrals. This has included meeting with school principals, providing program information for the school newsletters and presentations at Parents and Citizens meetings and school assemblies.

### Participants

The Go4Fun target population is children aged 7-13 years, with a body mass index (BMI) ≥85^th^ percentile and no co-morbidities, and have a parent/adult carer to accompany them to each session. Eligibility was assessed at the time of referral/contact with LHDs and based on anthropometric measures and a medical questionnaire completed by a parent/carer. Participation required written consent by a parent. The University of Sydney Human Research Ethics Committee approved this study.

### Up-scaling

A staged process for up-scaling was used with initial funding provided to employ program managers to deliver in two rural and regional LHD in July 2009, then funding to other LHDs to employ program managers was provided in a phased manner to enable the facilitation of the program across the state. The program was managed in each LHD however different models were used with some contracting private organisations such as Private Practice Dieticians, Divisions of General Practice and Aboriginal Medical Services to manage and deliver programs. Other LDH’s employed a program manager and contracted facilitators or allied health professionals to deliver programs.

### Recruitment

The prevalence of overweight/obesity is significantly higher among NSW children from low socioeconomic backgrounds [3], so these children and their families are the program’s primary target group. Because the program is delivered by a LHDs, knowledge of the community profile, expertise in engaging their communities and existing relationships with organisations working with the socially disadvantaged including Aboriginal communities are already established.

A number of strategies were undertaken to promote Go4Fun including advertising in newspapers and publications; attendance at community events with activities and distribution of promotional items (bouncy balls, wristbands); radio advertising; letterbox drops. Examples of Go4Fun advertising messages included “Free fun program for kids to become fitter, healthier and happier”; “Do you have children 7 to 13 years old? Are you worried about their weight?”; “Healthy, Active, Happy, Kids”; “Healthy lifestyle program for kids”; “Children and their families become fitter, healthier and happier”. Each LHD undertakes local recruitment utilising local media, engaging partnerships (with local non-government organisations, councils, school communities, youth clubs, recreational facilities, health services, private allied health services, and general practitioners) and providing workshops and professional development opportunities to raise program awareness.

Families may self-refer via a toll-free phone number, a text message or online registration to the program and secondary referrals were accepted from health professionals, organisations and community members. The top three referral sources accounting for the majority of referrals were (i) Schools (flyers, newsletters and school communications); (ii) Newspapers and local media articles and; (iii) Referrals from health professionals. Two other important referral sources were word of mouth and community groups; and local advertising in the community (letter box drops, posters, flyers etc.).

### Program description

Parent must accompany their child to the program so it was developed as a 20-bi-weekly (i.e. 10 weeks) after school program run during school terms however LHDs have adapted the program to 16, 18 or 20 sessions according to the timing of school terms, public holidays and to increase participant numbers in programs. It was left to the discretion of each LHD to decide which sessions to omit or modify. The theoretical basis of Go4Fun is to engage families in the process of weight management by addressing key components for individual-level behavioural change (education, skills training, and motivational enhancement) [8], and considering the need to engage multiple, interacting systems of influence within the family context [9]. Parental participation is central so at each session parents and children work together for the first hour, then children spend the second hour doing physical activity, while the parents undertake facilitated discussions. Nutrition sessions include healthy eating advice tailored for overweight/obese children; practical information on label reading and recipes; with weekly targets to achieve gradual behaviour changes. Behaviour change sessions aim to assist with difficulties in changing children’s habits and behaviours around eating and exercise. Sessions include goal and reward setting, problem solving, triggers and role modelling. Exercise sessions involve 1 hour of activities, undertaken on land or in water, progressively developing skills and building strength, fitness, confidence and self esteem.

### Program delivery

A range of health professionals are trained to deliver Go4Fun including: dieticians, nutritionists, exercise physiologists, physiotherapists, fitness leaders, social workers and health promotion officers and are delivered in a range of settings including community health centres, sport centres, and schools. LHD’s with high social disadvantage and with a high proportion of Aboriginal people, Aboriginal and welfare organisations such as the Aboriginal Medical Services, Aboriginal health workers and Anglicare are contracted to deliver programs often at their venues or the organisations referring participants into programs. Facilitators are required to complete two days of face-to-face training (including taking standard anthropometric measurements) plus online training facilitated by MEND Australia.

### Process evaluation

For process evaluation, we describe the child’s socio-demographic and risk factor profile, commensurate with the intended target group, the dose (i.e., number of sessions delivered and attendance rates) and reach (i.e., absolute number of participants and representativeness of individuals who participated). Facilitators enter participant data into the MEND Australia proprietary database.

At enrolment, parents completed a questionnaire on behalf of their child providing socio-demographic information including their child’s sex, date of birth, postcode of usual residence and Aboriginal status. Postcode of residence was used as a proxy for socio-economic status (SES), based on the Australian Bureau of Statistics’ Socio-Economic Indexes for Areas (SEIFA) and the Accessibility-Remoteness Index of Australia Plus (ARIA+) which determines geographical remoteness. SEIFA values were divided into quintiles (1 = most advantaged, 5 = least advantaged) and ARIA+measures were categorised as major city, inner regional, outer regional, remote/very remote [12,13].

At enrolment, facilitators measured children’s height (m) and weight (kg) for BMI (kg/m^2^), and waist circumference (cm) for waist-to-height ratio (WtHtr). Children’s BMI-for-age z-scores were calculated from the United States Centers for Disease Control and Prevention 2000 reference data [14]. WtHtr was categorised as <0.5 and ≥0.5, which is an indicator of central adiposity used to identify individuals “at increased metabolic risk” [15].

Upon completing the program parents and children participants were asked to complete a feedback survey that provided qualitative data on their experiences of the program.

### Analysis

Data analysis was undertaken in August-September 2013 using SPSS Complex Samples (version 19 for Windows, Chicago, IL, USA) to adjust standard errors and 95% confidence intervals (95% CI) for clustering of programs. All data collected between 2009 and 2012 were included in the analysis. Percent attendance was calculated, taking into account different program lengths (i.e., 16, 18 or 20 sessions) and program “completers” were defined as having attended at least 75% of sessions. Descriptive statistics describe socio-demographic and weight-related characteristics of participants. Sex differences, as well as differences between “completers” and “non-completers”, were determined by chi-square analyses for categorical variables and independent samples t-tests for continuous variables. Adjusted odds ratios (AOR) of the likelihood of children completing the program based on sex, Aboriginal status, ARIA+and SEIFA were derived from complex samples logistic regression. Statistical significance was accepted at P < 0.05.

## Results

Between July 2009 and December 2012, 296 Go4Fun® programs have been conducted in NSW. Children’s baseline socio-demographic and weight characteristics are presented in Table 1 and shows that 2,499 (54.8% girls; mean age 10.2 (1.7) years) overweight (28.9%) and obese (71.1%) children have participated in the program. One-third (36.7%) of participants resided outside of major cities in NSW, 76.5% were from socially disadvantaged areas (lowest three SEIFA quintiles) and 5.7% identified as Aboriginal.

**Table 1:** Characteristics of children (N = 2,499) enrolled in the NSW Go4Fun® program, 2009-2012

**Characteristics**	**N**	**Total**	**Girls**	**Boys**	**P-value**
n (%)	*2,499*		1,369 (54.8)	1,130 (45.2)	
Age (years; mean [SD])		10.2 (1.7)	10.1 (1.7)	10.2 (1.8)	0.09
**Weight status** (%)					1.0
Overweight		28.9	28.9	28.8	
Obese		71.1	71.1	71.2	
BMI z-score (mean [SD])		2.07 (0.41)	2.03 (0.42)	2.12 (0.38)	**<0.01**
WtHtr ≥0.5 (%)		94.5	93.4	95.8	**0.01**
**Aboriginal status** (%)					0.19
Non aboriginal		94.3	93.8	95.0	
Aboriginal or Torres Strait Islander		5.7	6.2	5.0	
**Residence (ARIA)** (%)	*2,472*				0.89
Major city		63.3	62.8	63.9	
Inner regional		25.0	25.5	24.4	
Outer regional		9.7	9.8	9.5	
Remote/very remote		2.1	1.9	2.2	
**Socioeconomic status (SEIFA index)** (%)	*2,472*				0.83
1st quintile (most advantaged)		5.9	5.8	5.9	
2nd quintile		17.7	17.1	18.4	
3rd quintile		25.2	26.1	24.2	
4th quintile		25.2	24.9	25.4	
5th quintile (most disadvantaged)		26.1	26.1	26.1	

*ARIA+*, accessibility remoteness index of Australia plus; *BMI*, body mass index; *WtHtr*, waist-to-height ratio; *SEIFA*, socio-economic index for areas.

**Figure 1:**
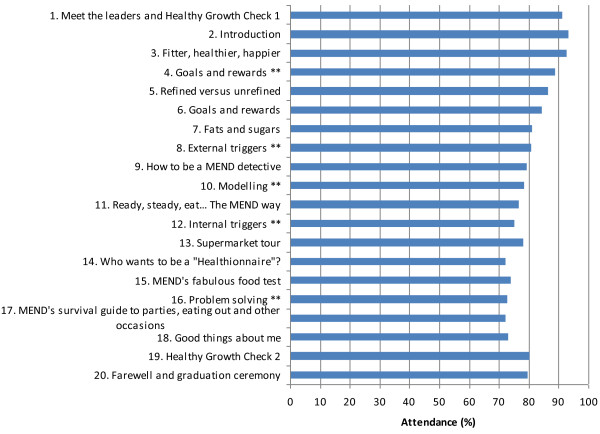
Attendance for children participating in 20 sessions, by program session (%)**.

More than half of children (57.9%) and their parents/carers attended at least 75% of sessions. Figure 1 lists the theme of each session and shows that overall, attendance at each program session was high for participants enrolled in 20-session programs. Table 1 shows the socio-demographic and weight characteristics of completers (based on 75% session attendance) and non-completers. Compared with completers, children who did not complete the program were more likely to be boys, Aboriginal children and participants from socially disadvantaged areas (three lowest SEIFA quintiles). After adjusting for sex (Table 2), Aboriginality, ARIA and SEIFA, girls were 1.2 (95% CI: 1.0, 1.4) times as likely to complete the program whereas Aboriginal children (AOR: 0.5; 95% CI: 0.4, 0.7) and participants from socially disadvantaged areas (three lowest SEIFA quintiles; AOR: 0.7; 95% CI: 0.6, 1.0) were less likely to complete the program.

**Table 2:** Characteristics of completers (attended ≥75% of sessions) and non-completers (attended <75% sessions)

**Characteristics**	**N**	**Completers**	**Non-completers**	**P-value**
n (%)	*2,499*	1,446 (57.9)	1,053 (42.1)	
Gender (% females)		56.8	52.0	**0.02**
Age (years; mean [SD])		10.1 (1.7)	10.2 (1.8)	0.13
**Weight status** (%)				0.85
Overweight		28.7	29.1	
Obese		71.3	70.9	
BMI z-score (mean [SD])		2.06 (0.40)	2.08 (0.42)	0.41
WtHtr ≥0.5 (%)		95.1	93.7	0.13
**Aboriginal status** (%)				**<0.01**
Non aboriginal		95.9	92.2	
Aboriginal or Torres Strait islander		4.1	7.8	
**Residence (ARIA)** (%)	*2,472*			0.14
Major city		62.4	64.6	
Inner regional		25.6	24.1	
Outer regional		10.5	8.5	
Remote/very remote		1.5	2.8	
**Socioeconomic status (SEIFA index)** (%)	*2,472*			**0.02**
1st-2nd quintile (most advantaged)		25.9	20.3	
3rd-5th quintile (most disadvantaged)		74.1	79.7	

*ARIA+,* accessibility remoteness index of Australia plus; *BMI*, body mass index; *WtHtr*, waist-to-height ratio; *SEIFA*, socio-economic index for areas.

Overall, participants’ feedback at the end of the program showed that 95.5% of parents indicated a good or very good level of satisfaction with the program and 87.1% of children indicated that they were either happy or very happy with the program. Some examples of parent statements included “I was sceptical at first but I even lost 4 kg”; “All around a very informative and useful program that we can implement for the whole family” and “It’s changed what we eat and our exercise habits”. Similarly some examples of the children’s statements included “The things we learnt were useful and we had lots of fun playing the games”; “The games and activities were very fun and made you fitter at the same time” and “Meeting up with new friends and getting fit in the process”.

## Discussion

In contrast to small-scaled interventions, process evaluation on large, up-scaled programs in the real world are limited. This is a significant evidence gap as the implementation of population-based intervention programs, such as Go4Fun®, in the real world face far greater challenges than the implementation of small efficacy trials that are controllable. More importantly, government decision-makers need information on a program’s strengths, weaknesses and where improvements can be made so that there is a continuous quality improvement process that underpins and justifies public funding of programs.

In terms of dissemination and implementation, the overall process evaluation findings reported here support the benefits of a significant government investment in a community-based child obesity treatment program to reach into socially disadvantaged communities, including Aboriginal and rural children. We found that the up-scaling of Go4Fun® into a real world setting was successful in its reach into Aboriginal families and families from lower socio-economic groups and more geographically remote locations where communities not only face significant greater health disadvantage, but limited access to health care services [16]. Almost 6% of participants identified as Aboriginal or Torres Strait Islander and given the significant health disadvantage of Aboriginal Australians [17,18], including higher rates of paediatric obesity [19], this finding was encouraging as the Go4Fun® program had not been specifically designed for Aboriginal communities and had a greater representation than would be expected from the community (NSW population prevalence of 5 to 15 year old Aboriginal children is approximately 4%) [20]. Aboriginal participants were however less likely to complete the program. This highlights the need for stronger collaboration with the Aboriginal community to ensure the cultural appropriateness of the program and to increase retention. This work has already commenced with 17 Aboriginal health workers being trained to deliver the program in LHDs where there are high populations of Aboriginal families.

There were some important lessons that we learnt during the up-scaling of Go4Fun across an entire jursidiction which assisted with the programs reach into socially disadvantaged families. These include working with local health promotion services to utilise their expertise in engaging with the local communities; supporting the program with comprehensive participant materials; ensuring that programs were delivered at a consistent location which enabled the program to become established in the community; and developing partnerships with key community members. A potential barrier of the program expressed by parents is the time commitment to attend twice weekly for 20 weeks.

Further, this process evaluation has provided important information which is necessary to program planning. For example, knowing that the children who did not complete Go4Fun were more likely to be boys, to be Aboriginal, and to come from lower quintiles of social advantage feeds into continued program improvement and development. That families from socially disadvantaged backgrounds were less likely to complete the program is congruent with the literature which shows that there is a clear social gradient in health and health literacy (22;23), but this information will inform program managers of the need to adapt Go4Fun® so that it resonates better with boys and socially disadvantaged families. Next steps are to conduct qualitative research through focus groups and individual interviews to understand why these population groups are less likely to complete the program.

Although the overall feedback from parents and children about the program was very positive, the twice a week time commitment was a consistent issue across all programs. In response, the Office of Preventative Health is currently piloting a randomised control trial examining the efficacy of using a once a week delivery mode for Go4Fun.

The importance of translational research for progressing public health and translating evidence from efficacy trials into practice has been emphasized [21]. Incumbent on the reporting of up-scaled programs is the need to report on the reach and representativeness of the targeted population. Reach is considered a fundamental aspect of scalability with few published studies extrapolating reach to those eligible in the population [22]. Compared with published findings of the UK MEND pilot program [9], Go4Fun® had greater reach into socially disadvantaged communities, suggesting that up-scaled programs, if appropriately funded and governed can reach into those families that are most at need. The two-tiered delivery model (i.e., central management by the NSW Ministry of Health and local management by LHDs) utilises the strengths of LHDs including local stakeholder relationships, knowledge of local systems and their communities.

The potential of community-based population-wide programs such as the Go4Fun® can only be realised when it is being used by a substantial proportion of the target audience. While a substantial number of overweight/obese children have participated in the Go4Fun® program, this represents an uptake of about 1.6% of overweight/obese children in NSW. There are opportunities to reach more families with overweight/obese children. The delivery of the program continues as part of the NSW Ministry of Health’s Healthy Children Initiative program of work. The Go4Fun® program could benefit from greater dissemination of program outcomes to health professionals and strengthening of clinical referral pathways to encourage secondary referrals; programs to increase parental awareness of unhealthy weight in childhood and; increased investment in state-wide advertising and marketing to promote awareness of the program and encourage self-referral to increase program reach. Next steps are to undertake the outcome evaluation of Go4Fun® and to assess the impact of different doses of attendance on program outcomes.

## Conclusions

The up-scaled and broadly delivered NSW Go4Fun® program achieved success in terms of reaching almost 2,500 overweight/obese children from socially disadvantaged backgrounds who potentially would not have accessed primary care or tertiary obesity treatment services. To date the program only reached a small proportion of overweight/obese children in the NSW population, highlighting the opportunity to increase efforts toward recruitment to the program and investigate modified program delivery to increase the program to retain socially disadvantaged families. Evaluation of the NSW Go4Fun® program both in terms of reach and effectiveness has national and international relevance, but more importantly has policy implications for the future roll out of the program, which in turn will impact on the effective expenditure of public funds.

## Abbreviations

ARIA+: Accessibility-remoteness index of Australia plus; BMI: Body mass index; CI: Confidence intervals; LHDs: Local health districts; MEND: Mind exercise nutrition do it; NSW: New South Wales; SE: Standard error; SEIFA: Australian bureau of statistics’ socio-economic indexes for areas (SEIFA); SES: Socioeconomic status; UK: United Kingdom.

## Competing interests

The authors declare that they have no competing interests.

## Authors’ contributions

DW and CIH designed the study. BN, BOH, LLH and AB conceptualized and designed the study evaluation. BN performed the statistical analyses. All authors participated in drafting the manuscript and revising it for important intellectual content. All authors read and approved the final manuscript.

## Pre-publication history

The pre-publication history for this paper can be accessed here:

http://www.biomedcentral.com/1471-2458/14/140/prepub
